# Optimized breeding strategies for multiple trait integration: III. Parameters for success in version testing

**DOI:** 10.1007/s11032-015-0397-z

**Published:** 2015-10-14

**Authors:** Xiaochun Sun, Rita H. Mumm

**Affiliations:** Department of Crop Sciences and the Illinois Plant Breeding Center, University of Illinois at Urbana-Champaign, 1102 S. Goodwin Ave., Urbana, IL 61801 USA; Dow AgroSciences, Indianapolis, IN USA

**Keywords:** Trait integration, Version testing, Computer simulation, Breeding strategy, Marker-assisted backcrossing, Equivalent performance, Success rate

## Abstract

Multiple trait integration (MTI) is the process by which a target hybrid (or variety) is converted to add value-added traits to the comprehensive performance package represented by that genotype. The goal is to recover all the attributes of the target hybrid, with the addition of the specified value-added traits. In maize, this process utilizes the backcross breeding method to incorporate transgenic events (or genes) of interest. Thus, MTI involves four main steps: single event introgression, event pyramiding, trait fixation, and version testing to ensure recovery of equivalent performance with at least one version of the converted hybrid. Based on a case study involving conversion of a target hybrid for 15 transgenic events (the female inbred parent was converted for 8 events, and the male inbred parent was converted for 7 events), we explored parameters in version testing to facilitate a high likelihood of recovering at least one version of the hybrid conversion with yield performance equivalency within 3 % of unconverted target hybrid. Using computer simulation, we explored the impact of two factors on the success rate of the MTI outcome: (1) the amount of residual NRP (non-recurrent parent) germplasm remaining in the converted hybrid and (2) the number of versions of each parental line conversion created. A range of residual NRP germplasm from 120 to 180 cM (which represents 95.0–96.6 % germplasm recovery of the target hybrid in maize) and up to 5 versions of each parental conversion were considered, with all possible hybrid combinations of each version of female and male RP (recurrent parent) conversion evaluated for yield. With 5 versions of each RP conversion and testing of 25 hybrid versions, a >95 % rate of success was realized when the amount of residual NRP germplasm in the hybrid conversion was ≤180 cM. When hybrid conversions contained ≤120-cM residual NRP germplasm, only 4 versions of one of the parental conversions were needed (rather than 5), requiring 20 versions of the hybrid conversion to be yield tested. These results have implications in the strategic design of an overall conversion program and for the upstream MTI process, especially in setting thresholds for the amount of NRP germplasm remaining in RP conversions. Furthermore, these results validate findings of (Peng et al. in Mol Breed 33:189–104, 2014a. doi:10.1007/s11032-013-9936-7, in Mole Breed 33:105–115, 2014b. doi:10.1007/s11032-013-9937-6) which outline effective breeding strategies to optimize earlier steps in MTI (preceding version testing).

## Introduction

Since the commercial release of the first transgenic crop cultivars in the mid-1990s, the role of genetically modified (GM) traits in the development of new and improved cultivars has expanded (Moose and Mumm 2008). Transformation has not only facilitated access to useful traits not otherwise available through crossing, these value-added traits have been rapidly adopted by farmers worldwide due to economic and environmental benefits offered (Brookes and Barfoot 2012), fueling the trend to include more and more GM traits in new cultivars. Today, new maize hybrids, especially those developed for North America, generally contain multiple transgenic events. SmartStax™ maize hybrids, for example, feature 8 different transgenes for various insect resistances and herbicide tolerances through the incorporation of 4 events (Monsanto Company 2007). By the year 2030, as many as 15–20 biotech traits may be routinely ‘stacked’ in new corn hybrids (Fraley 2012).

Multiple trait integration (MTI), the process of converting a target cultivar for multiple transgenic events (or other genetic factors) through backcrossing, is widely practiced in maize and other crops. This process typically consists of four steps: single event introgression, event pyramiding, trait fixation, and version testing which is defined as performance testing of various versions of a given target hybrid conversion (see Peng et al. 2014a for a detailed description of each step). Given the overarching aim of MTI to recover at least one version of the hybrid conversion with equivalent performance to the unconverted target hybrid along with stable expression of all the value-added traits, the version testing step is essential to achieving overall success in this process. If equivalent performance is not attained in the hybrid conversion, the product target has not been realized and commercial launch of the value-added cultivar may be delayed or canceled.

**Fig. 1:**
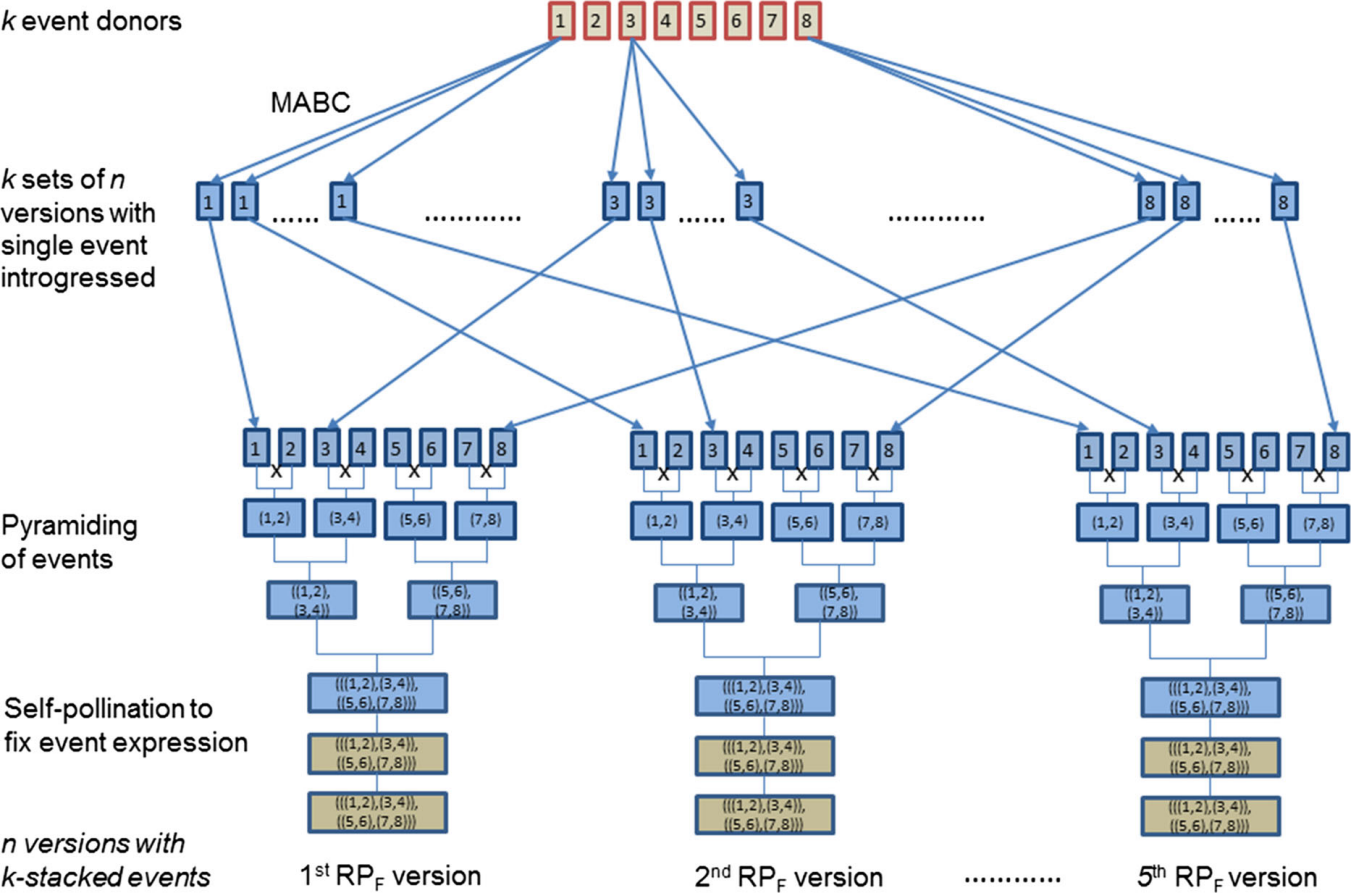
Process of generating *n* versions of a female recurrent parent (RP_F_) converted for *k* events (in this example, *n* = 5 and *k* = 8). Tan and *blue boxes* indicate homozygous and heterozygous states, respectively; *red* and *blue box outlines* signal differences in genetic backgrounds

In order to minimize the risk of failure to recover the target hybrid performance, typically multiple versions of the parental conversions are generated (Mumm and Walters 2001). This can be accomplished through parallel streams of backcrossing that originate with different BC1 families in single event introgression. Thus, *n* versions of each single event conversion of a given parent line ( Fig. 1), each containing the same transgenic event of interest, are produced. However, each version reflects a unique distribution of residual non-recurrent parent (NRP) germplasm from the trait donor. Versions of the parent line conversions are crossed in hybrid combination to produce various versions of the converted target hybrid, with the number of versions of the hybrid conversion being equal to the product of number of versions of the female RP conversion and the number of versions of the male RP conversion. The versions of the hybrid conversion are then typically evaluated as to performance, particularly yield performance, relative to the unconverted target hybrid to ascertain performance equivalency. In the converted hybrid versions, not only is distribution of NRP germplasm unique in the RP line conversions, the complementarity of the converted RPs is unique which may be important to the expression of heterosis for grain yield thought to be based mainly on dominant gene action.

This study builds upon work by (Peng et al. 2014a, b) to optimize MTI by identifying ‘best’ strategies and approaches for marker-assisted conversion involving a number of value-added traits. Whereas previous work concentrated on single event introgression, event pyramiding, and trait fixation in MTI, the present work focuses on version testing and recapturing yield performance equivalency within a strict range and with a high degree of probability. Success in version testing ultimately represents success in the entire MTI process. Although there have been numerous investigations to optimize breeding strategies in trait introgression (Hospital and Charcosset 1997; Hospital 2001; Visscher et al. 1996; Peng et al. 2014a, b), little has been published on the topic of version testing, despite its role and critical nature in determining the ultimate outcome of the MTI process.

In this study, we aimed to quantify the effect of the amount of residual NRP germplasm remaining in the converted hybrid and the number of versions of each parental line conversion created on the success rate, i.e., the probability of recovering at least 1 version of the hybrid conversion with equivalent performance with the unconverted target hybrid. Using computer simulation, the amount of residual NRP germplasm was varied from 120 to 180 cM (representing 96.6–95.0 % germplasm recovery of the target maize hybrid) and up to 5 versions of each RP conversion were produced. In addition, we considered ways to maximize efficiency in a breeding program by minimizing the number of versions of RP conversions to be created and the number of hybrid versions to be evaluated.

This present work utilizes a realistic breeding scenario (Peng et al. 2014a, b) that might be encountered in the seed industry involving the conversion of a target maize hybrid for 15 transgenic events. The breeding scenario assumes that (1) the transformation line is considered to be related to the female side of the heterotic pattern, (2) some events are required on the male side of the target hybrid; therefore, to balance out the number of events for introgression into each parent, 8 events will be introgressed into the female RP and 7 events into the male RP; (3) all events are new so conversions for each event are required; (4) events to be combined in a given RP are not linked genetically; (5) single event RP conversions containing 8–12 cM or less of NRP germplasm can be produced, with the caveat that ~1 cM or less of the residual NRP is in the 20-cM region flanking the event locus (as demonstrated in Peng et al. 2014a), and (6) hybrid conversions with a threshold of residual NRP germplasm of no more than 120, 140, 160, or 180 cM can be produced (using 15 single event RP conversions containing 8–12-cM residual NRP germplasm). Furthermore, we assumed that the hybrid targeted for conversion yields an average of 235.6 bushels per acre (14.79 metric tons per hectare) and we conservatively designated performance equivalency to be within 3 % (i.e., 7.1 bushels per acre or 0.44 metric tons per hectare).

## Materials and methods

### Genetic simulation

Computer simulations in this study were conducted in R statistical software (v2.10.1) (R Development Core Team 2011). Models of the genome and the MTI process followed Peng et al. (2014a, b) with two essential enhancements: representation of genetic architecture for grain yield (i.e., genes, QTL, and associated additive and dominance effects) and tracking residual NRP germplasm (i.e., size and location of fragments) remaining after completion of single event introgression in MTI through the event pyramiding and trait fixation steps.

The genome model for simulation was constructed according to the published maize ISU–IBM genetic map, with a total of 1788 cM (Fu et al. 2006). Events to be introgressed were assumed to be positioned on different chromosomes, avoiding issues related to linkage. That is, the 8 events introgressed into the female RP were arbitrarily assigned to Chromosomes 1, 3, 4, 5, 7, 8, 9, and 10; and the 7 events introgressed into the male RP were arbitrarily assigned to Chromosomes 1, 2, 3, 4, 6, 8, and 9. Event placement within the chromosome was random. Furthermore, events were assumed to be traceable by a single (perfect) marker and no genetic variation was considered for the event expression.

Equivalent performance between the converted and unconverted target hybrid was based on grain yield, assumed in our simple model to be controlled by 100 QTL randomly positioned across the genome. Additive and dominance effects were assigned to QTL in accord with the method by Sun and Mumm (2015) based on distributions of additive and dominance effects derived from meta-analyses of previously published QTL studies. A QTL was assumed to be a gene cluster with 5 genes spanning 1 cM in total with a distance of 0.2 cM between genes. All genes and markers were assumed to be bi-allelic and informative (polymorphic between RPs), thus, resulting in a QTL represented by a multi-allelic haplotype. In the computer simulation, genetic markers were evenly spaced across the 10 chromosomes of maize at 0.2-cM interval (8950 markers in total across the genome) for use in tracking QTL (and genes) associated with yield.

### Modeling heterosis

We further assumed that alternate alleles were fixed on opposite sides of the heterotic pattern through generations of selections to maximize heterosis; in other words, heterosis was assumed to be purely caused by dominance effects. Prior to introgression, alleles from male and female side were set to ‘*G*’ and ‘*g*,’ respectively. However, residual NRP germplasm resulting from the conversion process may lead to certain loci with homozygous (e.g., *GG* or *gg*) status instead of heterozygous in the converted hybrid, particularly in cases where the transformant line used to generate the event originated from a heterotic group different than one of the RPs. In our breeding scenario, all events were assumed to be generated in a female-type transformant line, so the possibility exists for male RP conversions to harbor some germplasm from the female heterotic group.

Although heterosis is primarily explained by two important theories, dominance and overdominance (Birchler et al. 2003), in this study we adopted the suggestions from Edwards et al. (1987) who attributed QTL overdominance effects detected in maize yield to the repulsion phase linkage of several genes with partial dominance. Specifically, additive effects of genes sampled from the normal distribution with null mean were automatically assigned positive or negative sign, indicating random repulsion phase linkages between genes. The positive mean of dominance coefficients (i.e., 0.152; see Sun and Mumm 2015) fit with overall partial dominance gene action and findings of Kacser and Burns (1981) that dominance coefficients tend to have a positive direction.

### Genotypic values

Equivalent performance between the converted and unconverted target hybrid was tracked by the genotypic value, which was estimated according to Cockerham (1954):

1 $${\text{Geno}}_{i} = \sum\limits_{k = 1}^{N} {u_{ik} \alpha_{1k} + v_{ik} d_{k} } ,$$

where ${\text{Geno}}_{i}$ was the *i*th individual hybrid and design elements $u_{k}$ and $v_{k}$ were defined according to the traditional $F_{\infty }$ model as

$$u_{k} = \left\{ {\begin{array}{*{20}c} {\begin{array}{*{20}c} 1 & {GG} \\ \end{array} } \\ {\begin{array}{*{20}c} 0 & {Gg} \\ \end{array} } \\ {\begin{array}{*{20}c} { - 1} & {gg} \\ \end{array} } \\ \end{array} } \right.\quad {\text{and}}\quad v_{k} = \left\{ {\begin{array}{*{20}c} {\begin{array}{*{20}c} 0 & {GG} \\ \end{array} } \\ {\begin{array}{*{20}c} 1 & {Gg} \\ \end{array} } \\ {\begin{array}{*{20}c} 0 & {gg} \\ \end{array} } \\ \end{array} } \right.,$$

and parameters $\alpha_{1k}$ and $d_{k}$ were additive and dominance effects of *k*th gene. Additive effects $\alpha_{1} \left( {\alpha_{2} } \right)$ defined as the effect of homozygote carrying the allele from male (female) were drawn from a normal distribution $N\left( {0,\;0.044\sigma_{p}^{2} } \right)$ (per Sun and Mumm 2015), where $\sigma_{p}$ was the phenotypic standard deviation of inbred parents. $\alpha_{1k}$ was used in (1) because $u_{k} = 1$ when male allele ‘*G*’ was homozygous. And dominance effects, *d*, were obtained from the product of homozygous effects (*a*) defined as half of the difference between two homozygotes ($a = \frac{{\alpha_{1} - \alpha_{2} }}{2}$) (Falconer and Mackay 1996) and the dominance coefficients (${d \mathord{\left/ {\vphantom {d a}} \right. \kern-0pt} a}$ ratio) which were sampled from a normal distribution $N\left( {0.152,0.392} \right)$ (per Sun and Mumm 2015).

The genotypic variance was calculated as follows

2 $$V_{G} = V_{A} + V_{D} = \sum\limits_{k = 1}^{N} {\sigma_{{a_{k} }}^{2} + \sum\limits_{k = 1}^{N} {\sigma_{{d_{k} }}^{2} } } ,$$

where *N* was the number of gene loci, and $\sigma_{{a_{k} }}^{2} {\text{ and }}\sigma_{{d_{k} }}^{2}$ were additive and dominance variance for *k*th locus. In case of the hybrid population derived from two inbred lines, the allele frequency was 0.5 for all segregating genes and the additive and dominance variance of a single locus became $\sigma_{a}^{2} = \frac{1}{2}a_{{}}^{2} \;{\text{and}}\;\sigma_{d}^{2} = \frac{1}{4}d_{{}}^{2}$ (Falconer and Mackay 1996), leading (2) to

3 $$V_{A} = \frac{1}{2} \times \sum\limits_{k = 1}^{N} {a_{k}^{2} } \quad {\text{and}}\quad V_{D} = \frac{1}{4} \times \sum\limits_{k}^{N} {d_{k}^{2} } .$$

### Phenotypic hybrid mean and standard deviation

Simulated hybrids were considered to have an expected mean performance (±standard deviation) for grain yield of 235.6 (±48.1) bushels per acre [14.79 (±3.02) metric tons per hectare], which was computed as follows. The target hybrid performance (*H*) is a function of mid-parent value and heterosis which was assumed to be purely caused by dominance effects. Thus, expected target hybrid performance $E(H) = \mu_{p} + N \times E(d)$, where $\mu_{p}$ was the mean of two inbred parents, *N* was the number of gene locus and $E(d)$ was the mean of dominance effect. Given the distribution of additive effects and dominance coefficients, $E(d) = E\left( {a \times \frac{d}{a}} \right) = E(a)E\left( {\frac{d}{a}} \right) = 0.152 \times E(a)$, where *a* was the homozygous effect following a truncated distribution of additive effects, i.e., $a\;\sim\;TN\left( {0 ,0.044 \times \sigma_{p}^{2} } \right)$, with truncation point at 0. And $E(a) = \int\limits_{0}^{\infty } {af(a) = } \int\limits_{0}^{\infty } {\frac{2a}{{\sqrt {2\pi } \sigma }}e^{{\frac{{ - a^{2} }}{{2\sigma^{2} }}}} } da = \sigma \sqrt {\frac{2}{\pi }}$, where $f(a)$ was the probability density function of *TN* and $\sigma$ was the standard deviation of additive effects, i.e., $\sigma = \sqrt {0.044} \sigma_{p}$. Mean and standard deviation of inbred population,$\mu_{p}$ and $\sigma_{p}$ were set to 70 and 13 bushels per acre (4.40 and 0.82 metric tons per hectare), respectively, based on average values of grain yield performance of 12 elite inbred lines representing important heterotic subgroups in US maize commercial germplasm (Mansfield and Mumm, unpublished data). Given the above values, the expected hybrid was $E(H) = \mu_{p} + N \times E(d) = 70 + 500 \times 0.152 \times \sqrt {\frac{2}{\pi }} \sigma = 235.6$ bushels per acre.

Based on (3), the expected additive and dominance are $E(V_{A} ) = 0.5 \times 500 \times E(a^{2} )$, and $E\left( {V_{D} } \right) = 0.25 \times 500 \times E\left( {d^{2} } \right) = 125 \times E\left( {a^{2} } \right)E\left( {\left( {\frac{d}{a}} \right)^{2} } \right)$, where $E\left( {a^{2} } \right) = E\left( {\left( {\frac{{\alpha_{1} - \alpha_{2} }}{2}} \right)^{2} } \right) = \frac{1}{2}E\left( {\alpha_{1}^{2} } \right)$ (Falconer and Mackay 1996). Therefore, $E\left( {V_{A} } \right) = 125 \times 0.044 \times 13^{2} = 925.5$, and $E\left( {V_{D} } \right) = 125 \times \frac{{0.044 \times 13^{2} }}{2} \times 0.392 = 182.2$ bushels per acre. Estimating narrow sense heritability (*h*^*2*^) for grain yield at 0.4, the expected error variance is $E\left( {V_{e} } \right) = 1.5E\left( {V_{A} } \right) - E\left( {V_{D} } \right) = 1212.1$. And the total phenotypic standard deviation of the hybrid is $\sqrt {V_{A} + V_{D} + V_{e} }$ = 48.1 bushels per acre (or 3.02 metric tons per hectare).

### Representation of differences between the versions of a conversion

In this study, we simulated introgression of 15 events into a target elite hybrid, integrating 8 and 7 events into female and male inbred parents, respectively. Since each version of a RP conversion has at most 8–12 cM of residual NRP remaining in its genome, a version of the female RP containing all 8 events will contain at most 64–96-cM residual NRP. The variations between versions of the RP thus arise from different distributions of the residual NRP. Note that the number of versions of each single event RP conversion was the same as the number of versions of the multi-event RP conversion, which in this study was 5. We modeled the size and location of NRP fragments resulting from recombination and selection during MTI in accordance with the results obtained by Peng et al. (2014b) as to the amount of residual NRP germplasm.

The length of residual NRP germplasm segments, expressed in terms of units of genetic distance (cM), at the conclusion of single event introgression was assumed to follow an exponential distribution. Since the smallest NRP segment was always larger than zero, we fitted a truncated exponential distribution to the data. The density for the truncated distribution was

4 $$P\left( {y_{i} |\lambda } \right) = \frac{{\lambda e^{{ - \lambda y_{i} }} }}{{\int_{c}^{\infty } {\lambda e^{ - \lambda y} } {\text{d}}y}} = \lambda e^{{ - \lambda \left( {y_{i} - c} \right)}} ,$$

where *y*_*i*_ was *ith* observed data, *c* was the truncation point, and $\lambda$ was the rate. Referring to the previous introgression results (Peng et al. 2014a, b), the minimum size of NRP segments in the 20-cM flanking regions bracketing events was 1 cM (i.e., *c* = 1). To draw sample *Y* from the truncated distribution, we simply simulated $X \sim {\text{EXP}}\left( {\hat{\lambda }} \right)$, where $\hat{\lambda }$ was obtained by taking the reciprocal of expected genetic length of non-flanking regions defined as NRP residing outside of the 20-cM regions flanking the event of interest and further took $Y = X + 1$.

To represent pyramiding of the single events in a specific RP, we repeated the above sampling procedure over *k* times, where *k* was the total number of events to be introgressed. The above procedure was based on assumptions that the introgression of one event was independent of others and that the pyramiding process contributed little to genetic content shuffling. When introgressing 15 events into the target hybrid, with 8 events to a female line and 7 events to a male line, the total amount of residual NRP in a hybrid conversion was set to a series of values: 180, 160, 140, and 120 cM, which corresponds to 10, 8.9, 7.8, and 6.7 % proportion on NRP germplasm across the genome, respectively. Thus, for each event in the converted target hybrid, an average of 12, 10.6, 9.3, and 8 cM, respectively, was associated with each single event conversion, 1 cM of which was located in the region flanking the event locus and the rest located at other genomic locations across the genome. The 1-cM-length fragments in the regions flanking the event loci were randomly positioned within this 20-cM region in the simulation, and the balance of the NRP fragments were randomly placed across the rest of genome in accord with (4). *In silico* female (male) inbred versions were thus generated by repeating the above sampling-positioning procedure *n*_f_ (*n*_m_) times, where *n*_F_ (*n*_M_) was the number of female (male) versions. To stay within realistic bounds, the analysis considered *n*_F_(*n*_M_) ≤ 5 to find the optimal number of inbred versions on each pedigree side. Versions of a target hybrid conversion were created by crossing *n*_F_ × *n*_M_ versions of the parental conversions, respectively.

We also examined whether it was possible to identify the ‘best’ versions of the RP conversions, i.e., those not having yield genes indigenous to the target RP replaced by residual NRP germplasm. Genotypic values were estimated for the versions of the target hybrid conversion to provide a ranking of the ‘best’ RP versions.

This simulation scenario (including creating genetic map, positioning QTL, modeling genetic effects, generating versions of the RP conversions, and estimating genotypic values of versions of the hybrid conversion) was repeated 1000 times to calculate the average success rate, which in this study is the likelihood of covering at least one hybrid conversion with equivalent performance within 3 %. To calculate the mean and standard errors of the success rate, the whole procedure was also repeated six times.

## Results and discussion

Since version testing is the ultimate determination of the success of the MTI process and the achievement of a given product (cultivar) target, the ideal success rate would be 100 %. In this case, the MTI process would, without fail, result in recovery of at least one version of the target hybrid conversion with equivalent performance. In reality, a somewhat lower success rate of 95 % might be considered an acceptable and realistic threshold.

Results of computer simulation indicated that a ≥95 % success rate is achievable within the range of 120–180 cM of residual NRP germplasm in the converted hybrid using ≥5 versions of the female and male parent conversions (Table 1). Specifically, with 5 versions of each parent conversion and performance testing of all 25 versions of the hybrid conversion, the success rate ranged from 96.60 to 95.18 %, respectively, as the amount of residual NRP germplasm increased from 120 to 180 cM in the converted hybrid. However, as the amount of residual NRP germplasm in the converted hybrid increased, the success rate steadily decreased.

**Table 1:** Estimated success rate of recovering ≥1 version of the target hybrid conversion with yield equivalency within 3 % across a range of amounts of residual NRP germplasm in the hybrid conversion, based on performance testing of all possible hybrid combinations of various versions of both hybrid parent conversions

NRP germplasm in converted hybrid (%)	Germplasm recovery of the target hybrid (%)	Total residual NRP germplasm (cM)	NRP for each single event conversion (cM)	No. female parent versions	No. male parent versions		
					3 (%)	4 (%)	5 (%)
6.7	96.6	120	8.0	3	88.82	92.23	93.68
				4	92.20	93.73	95.05
				5	93.25	95.20	96.60
7.8	96.1	140	9.3	3	88.73	91.93	92.58
				4	90.82	93.13	94.67
				5	93.05	94.88	95.87
8.9	95.5	160	10.7	3	88.15	90.85	91.92
				4	90.70	93.02	94.45
				5	92.30	94.73	95.32
10	95.0	180	12.0	3	87.13	90.73	91.18
				4	90.50	92.57	93.75
				5	92.03	93.97	95.18

Values were obtained from 1000 simulations with six repeats. Standard errors of the estimates were 0.56–0.59 %

The relationship between NRP% and Germplasm Recovery% is given by ${\text{NRP}}\left( \% \right) = \left( {100 - {\text{GR}}\% } \right) \times 2$

In general, the success rate was positively correlated with the number of versions of the RP conversion (Table 1). Across a range of 120–180 cM of NRP germplasm remaining in the hybrid, 5 versions of each parental conversion were associated with a ≥95 % success rate (Table 1); 4 versions of each parental conversion were associated with a <94 % success rate; and 3 versions of each parental conversion were associated with a <89 % success rate. In each of these cases, the success rate assumes that all possible hybrid combinations of versions of parental conversions were evaluated in yield performance trials. In this specific range of residual NRP germplasm, an approximate 8 % increase in success rate was observed as the number of parental conversions on each side of the pedigree was increased from 3–5.

In the particular case of ≤120-cM NRP germplasm remaining in the converted hybrid, a ≥95 % success rate can be achieved with 5 versions of one parental conversion and 4 of the other. With 5 versions of the male parent conversion and 4 versions of the female parent conversion, the success rate averages 95.05 %; with 4 versions of the male parent conversion and 5 versions of the female parent conversion, the success rate averages 95.20 %. Statistically, there is no difference in these likelihoods of success (given the magnitude of standard error) despite the fact that 8 events were introgressed into the female parent and only 7 into the male parent. This was balanced somewhat by the fact that male conversions are somewhat riskier given the assumption that the transformant line used to generate the events was related to the female heterotic group, meaning that residual NRP germplasm in the male parent conversion can dilute expression of heterosis in the converted hybrid. The potential for reduction in the number of parental versions per conversion has important implications for resource allocation in the conversion program. Furthermore, with the reduction in the number of versions of one parent also comes a reduction in the number of hybrid combinations to be yield tested, i.e., performance testing of 20 versions of the hybrid based on combination of the 4 + 5 versions of the parental conversions.

We explored the potential for reducing the number of hybrid versions to be tested even further by identifying a ‘best’ subset of parental versions using estimated genotypic values. Versions of the converted hybrid were ranked based on genotypic value to assess the ‘best’ 3 versions of each RP, i.e., those not having yield genes indigenous to the target RP replaced by residual NRP germplasm. However, identifying the ‘best’ 3 versions from up to 5 versions of each parent and then testing 9 versions of the converted hybrid did not result in achieving the 95 % success rate threshold across the range of residual NRP germplasm considered. Table 2 displays the estimated success rates of recovering ≥1 hybrid conversion with yield equivalency within 3 % based on performance testing of 9 hybrid combinations of versions of RP conversions after selecting the ‘best’ 3 versions of each RP from the total number of versions created. Looking particularly at the case involving ≤120-cM residual NRP germplasm in the converted hybrid and creation of 5 versions of the female conversion and 4 versions of the male conversion, when the number of selected versions based on breeding value are reduced to 3 for each parent, the success rate is 93.11 %, which falls short of the 95 % threshold by nearly 2 % points.

**Table 2:** Estimated success rate of recovering ≥1 version of the target hybrid conversion with yield equivalency within 3 % based on performance testing of 9 hybrid combinations of versions of the RP conversions after selecting the ‘best’ 3 versions of each RP from the total number of versions created

Total residual NRP germplasm (cM)	No. female versions	No. male versions		
		3 (%)	4 (%)	5 (%)
120	3	88.82	90.45	91.62
	4	90.42	91.67	92.96
	5	91.20	93.11	94.47
140	3	88.73	90.16	90.55
	4	89.06	91.08	92.58
	5	91.00	92.80	93.76
160	3	88.15	89.10	89.89
	4	88.95	90.97	92.37
	5	90.27	92.65	93.22
180	3	87.13	88.98	89.18
	4	88.75	90.53	91.69
	5	90.01	91.90	93.09

Values were obtained from 1000 simulations with six repeats. The standard errors of estimates were 0.51–0.57 %

In general, the success rate dropped approximately 2 % when testing a subset of 9 hybrid versions based on the 3 versions of each RP ranked highest for genotypic value. In real life, this reduction could be even more for a lowly heritable trait like yield since, in this present study, we used true genotypic value rather than estimated breeding value to serve as the selection index. In short, with this approach to cut the number of hybrid versions tested to 9 in total, not only is the threshold success rate not achieved but additional genotyping costs associated with genomic selection among finished conversions would be incurred in a conversion program as well. It may be more reasonable to conduct genomic selection during single event introgression, which would simply involve a denser marker set to select for RP germplasm recovery (Peng et al. 2014a used 100 markers to select for RP germplasm recovery), not only to identify those progeny with the least NRP germplasm overall but to assess the positional effects of NRP fragments in the genome as well.

In conclusion, version testing is implemented in a trait integration program to identify at least 1 version of the target hybrid conversion that satisfies both requirements of incorporating all the transgenic events (or genes) of interest and recovering equivalent performance of the target hybrid. Unless these criteria are met, the MTI may not achieve commercial reality and all efforts may have been in vain. While failure does not generally mean a complete restart, it would likely result in significant delay of perhaps multiple years in commercial introduction of the new value-added cultivar.

Our results highlight the vulnerability and risk level associated with a conversion program. Even with reduction of total NRP germplasm in the hybrid conversion to as little as 6.7 % or less and 5 versions of each parent conversion and testing of 25 versions of the converted hybrid with 96.6 % recovery of the target hybrid germplasm, there is an estimated success rate of 96.60 %. While this success rate is reasonable, even encouraging, any room for failure is difficult to reconcile given the resources and time expended in MTI. The importance of strategic design and optimization of a conversion program for greatest efficiency is emphasized.

We used computer simulation to explore and develop some guidelines and approaches for such a program. The value of computer simulation in guiding critical decisions facing the plant breeder is enhanced if the underlying models such as the genome model accurately portray real genetic processes and true genetic architecture (Sun et al. 2011). In the present study, we used distributions of additive and dominance effects which were derived from meta-analyses of previously published QTL studies (Sun and Mumm 2015) to update the genome model used to simulate genetic effects. This aided us in estimating the positional effects of NRP germplasm remaining in finished conversions of the target hybrid improved for 15 events relative to recovery of yield performance equivalency.

These results have implications in the strategic design of an overall conversion program and for the upstream MTI process, especially in setting thresholds for the amount of NRP germplasm remaining in RP conversions. Our results suggest that such thresholds for single event introgression are optimal at levels ≤(1/*k*) × 120 cM, where *k* is the number of events to be stacked in the target hybrid. Furthermore, these results validate findings of Peng et al. (2014a, b) which outline effective breeding strategies to optimize earlier steps in MTI (preceding version testing).

## Abbreviations

cM: centiMorgan
MTI: Multiple trait integration
NRP: Non-recurrent parent
QTL: Quantitative trait locus (loci)
RP: Recurrent parent
RP_F_: Female recurrent parent
